# Field test of Easter lilies transformed with a rice cystatin gene for root lesion nematode resistance

**DOI:** 10.3389/fpls.2023.1134224

**Published:** 2023-03-24

**Authors:** Becky Westerdahl, Lee Riddle, Deborah Giraud, Kathryn Kamo

**Affiliations:** ^1^ Department of Entomology and Nematology, University of California, Davis, Davis, CA, United States; ^2^ Easter Lily Research Foundation, Brookings, OR, United States; ^3^ University of California Cooperative Extension, Eureka, CA, United States; ^4^ Floral & Nursery Plants Research Unit, United States Department of Agriculture (USDA), Beltsville, MD, United States

**Keywords:** *Pratylenchus penetrans*, cysteine protease, *Lilium longiflorum*, nematode management, pesticide use

## Abstract

Easter lilies, *Lilium longiflorum* cv. Nellie White are a staple of the floral industry. In the U.S. most of the Easter lilies are grown in Oregon and California along the coast where there is a micro climate that is favorable to growth of lilies. The main pest when growing lilies in the field is *Pratylenchus penetrans*, the root lesion nematode. Easter lilies are one of the most expensive crops to produce because of the cost of chemicals used to control *P. penetrans* and other pathogens that infect the lilies. Our previous study had shown that transgenic Easter lilies containing a rice cystatin gene (Oc-IΔD86 that has a deleted Asp86) were resistant to *P. penetrans in vitro*. This study examined growth characteristics of five independently transformed lines of the cystatin Easter lilies compared to non-transformed Nellie White for three seasons in the field in Brookings, Oregon. Liles grown in three soil chemical treatments 1) preplant fumigation, 2) preplant fumigation plus at plant organophosphate, and 3) at plant organophosphate were compared to those grown in nontreated soil. Growth characteristics evaluated included: time of shoot emergence, survival of plants, size of plants, visual ratings of plant health, basal roots and stem roots, weight of foliage and roots, and number and size of bulblets that developed on stems. Nematodes were counted following their extraction from the roots. While not totally resistant, when planted in the field, transformed lines demonstrated and maintained a degree of resistance to lesion nematode over two growing seasons and displayed desirable growth and quality characteristics similar to non-transformed lilies.

## 1 Introduction

*Pratylenchus penetrans*, the root lesion nematode, is a major pest that ranks third for the economic damage that it causes to crops worldwide (Davis and MacGuidwin, 2000). It is a particular problem in the Pacific Northwest where it infects Easter lilies (Westerdahl et al., 2003; Westerdahl et al., 2020). The wholesale value of Easter lilies is $24 million, and their cultivation impacts 890 hectares of land, 320 U.S. greenhouse growers and countless retailers (USDA Floriculture Crops 2015 Summary). The main pest threatening lily production is *P. penetrans* which can reduce size and quality of the plant by feeding on its roots. There are no known cultivated lily species resistant to *P. penetrans*. Currently soil fumigation and other pesticide applications directed against this nematode pest costs growers $3,840/0.41 hectare.

Effective nematode management requires a combination of clean planting stock and clean soil, as well as an understanding of the biology of the pests involved. Lily growers are very interested in alternatives to pesticides because of their effect on human health, production costs, and their anticipated removal from the market. In the Easter lily cropping system, severe pest pressure resulting from both nematode infested soil and infected planting stock results in growers using a dual nematicide application consisting of a dual preplant fumigant treatment followed by an organophosphate at planting (Westerdahl et al., 2003; Westerdahl et al., 2020).

Cystatins are proteinase inhibitors that interfere with digestion of protein in various nematode species resulting in nematodes that have delayed development. The rice cystatin (oryzacystatin), Oc-IΔD86, has a deleted Asp86. Hairy roots of tomato transformed with this gene were found to have resistance to *Globodera pallida*, the potato cyst nematode as the female nematodes were smaller and had decreased fecundity (Urwin et al., 1995). This variant cystatin Oc-IΔD86 was compared to the intact rice cystatin lacking the amino acid deletion, and tomato roots with Oc-IΔD86 were more effective for *G. pallida* resistance (Urwin et al., 1995). Effective resistance to *G. pallida* in potato plants transformed with Oc-IΔD86 was demonstrated in a field study (Urwin et al., 2001). Rice plants transformed with Oc-IΔD86 showed a 55% reduction in egg production by *Meloidogyne incognita*, root-knot nematodes after growing rice plants 42 days in nematode-infested soil in pots (Vain et al., 1998). Transgenic *Arabidopsis* plants containing Oc-IΔD86 were resistant to the migratory nematode *Rotylenchulus reniformis* (the reniform nematode) and to two sedentary nematode species, *Heterodera schachtii* (beet-cyst nematode) and *M. incognita* (root-knot nematode) in a greenhouse study (Urwin et al., 1997; Urwin et al., 2000). Resistance against *Radopholus similis* was achieved in transgenic Cavendish banana plants expressing Oc-IΔD86 when grown in the greenhouse (Atkinson et al., 2004).

Cystatins other than Oc-IΔD86 have been shown to affect migratory nematodes. Alfalfa plants containing either the rice oryzacystatin I or II genes showed resistance to *P. penetrans* when plants were growing in sterile conditions in a growth chamber (Samac and Smigocki, 2003). Sweetpotato plants transformed with the oryzacystatin-I gene showed resistance in the field to *Ditylenchus destructor* (stem nematodes) (Gao et al., 2011). Plantains (*Musa* spp.) were transformed with either maize cystatin which is a cysteine proteinase and/or a synthetic peptide that interferes with chemoreception of the nematode (Roderick et al., 2012). The highest level of resistance against *R. similis* was in plantains with the maize cystatin (84% resistance), followed by dual defense genes (70% resistance), and then the synthetic peptide (66%) in screen house trials. Select lines of the transgenic plantains were grown in the field in Uganda where the main nematode pests are *R. similis* and *Helicotylenchus multicinctus* (Tripathi et al., 2015). The highest resistance was found in transgenic plantains with the synthetic peptide or dual defense genes, and several transgenic plant lines have been selected for further evaluation of plant vigor, yield, root necrosis and death, and toppling of the plants.

Field grown Easter lily bulbs are sold to greenhouse operations nationwide for forcing to produce flowering plants at Easter. Bulbs are grown for two to four years before they are large enough for sale. Typically, land is prepared in May, fumigated in July, bulblets are planted from August through October, and bulbs are harvested the following August through October (Roberts et al., 1985). Planting stock can be from immature bulbs, individual scales from a bulb, or bulblets that develop on the underground portion of the stem intermingled with stem roots. Roots grow both on the below ground portion of the stem and from the base of the bulb. Bulbs not reaching marketable size are replanted for an additional year.

**Figure 1:**
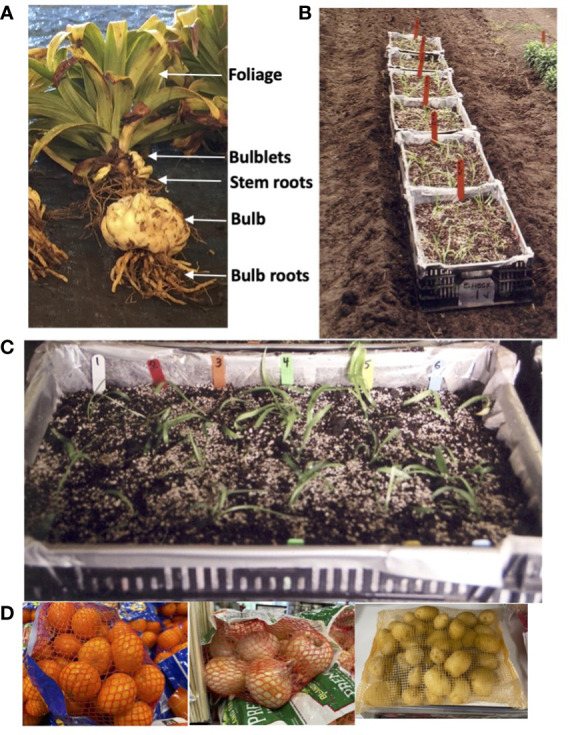
**(A)** View of a mature Easter lily plant indicating location of the parts analyzed in the trials: foliage (visually rated), bulblets (counted and weighed), stem roots (visually rated), bulb (weighed), and bulb roots (visually rated). **(B)** View of perforated plastic crates used in Trial 1. To be certain that all plantlets could be recovered, we modified a standard technique used in the greenhouse of planting in perforated plastic crates and then planted the crates in the field. The crates were placed in flat bottomed furrows in the field either in nontreated soil or in soil that had been previously fumigated. Soil from the same treatment was later filled in around the crates. Photo is of the six crates placed in untreated soil. Half of the crates were later treated with an organophosphate. **(C)** View of a single plastic crate with three plants of each lily type planted in a row identified by colored stakes: NTrNW (white), CYS 11 (red), CYS 25 (orange), CYS 55 (green), CYS 59 (yellow) and CYS 71 (blue). **(D)** Mesh bags of the type used to hold plants and bulbs for individual replicates prior to dipping in a preplant fungicide bath and then planting in the field.

Over a period of more than 40 years of trials, rating and measurement systems have evolved for the different parts of an Easter lily plant (**Figure 1A**). From top to bottom, the parts of the plant utilized in ratings and measurements are the foliage, belowground stems that emerge from the bulb or bulblet that was planted, stem roots that grow on the belowground stems, bulblets that develop amongst the stem roots, the bulb or bulblet that was planted, and basal roots growing from the base of the bulb or bulblet.

In three field trials, growth characteristics of *Lilium longiflorum* cv Nellie White plants that had been propagated *in vitro* (non-transformed, NTr), and five lines (CYS 11, CYS 25, CYS 55, CYS 59, CYS 71) of Easter lilies transformed with the Oc-IΔD86 gene were planted and evaluated in three soil chemical treatments 1) preplant fumigation, 2) preplant fumigation plus at plant or post plant organophosphate, and 3) organophosphate alone; and compared to those grown in nontreated soil to see if they could provide an improvement over or replace the standard soil treatments.

## 2 Materials and methods

Trials were conducted at the Easter Lily Research Foundation (ELRF) Station in Brookings, OR, in a field managed to provide a uniform population of *P. penetrans* by rotating lilies with clover. Tractor drawn implements were used for land preparation and bed formation and was done with great precision to ensure the integrity of the individual plots. Because several years of pasture rotation are practiced between crops, the initial soil population of *P. penetrans* at planting of trials is typically at a level that is not detectable by standard soil extraction techniques. However, the levels present combined with low levels of nematodes in planting stock are sufficient to cause significant damage at harvest (Westerdahl et al., 2003).

### 2.1 Trial 1: May 2014 to September 2014

*Lilium longiflorum* ‘Nellie White’ (NW) were transformed with the rice cystatin gene Oc-IΔD86 under control of the CaMV 35S promoter as previously described (Vieira et al., 2015). The lilies were developed from NW field planting stock obtained from Dahlstorm and Watt Bulb Farms in Smith River, CA. Transgenic lilies growing *in vitro* were shipped to the Easter Lily Research Foundation in Brookings, Oregon (Curry County) under an APHIS interstate transport permit 436422. The field release permit was 14-056-103n.

We expected to receive plantlets in the fall of 2013 to plant for the 2013 to 2014 growing cycle. but the plants were not available until May of 2014. Because the ability of the laboratory raised plantlets to survive under field conditions had never been tested, the decision was made to proceed with Trial 1 to determine if the plantlets could survive when removed from tissue culture and planted in the field. To be certain that all plantlets could be recovered, we modified a standard technique used in the greenhouse of planting in perforated plastic crates (**Figure 1C**) and then planted the crates in the field (**Figure 1B**). Once planted, the crates were placed in flat bottomed furrows in the field and surrounded with soil. The plants not planted in the field were grown in plastic boxes in the greenhouse until fall when they were recovered and planted in the field for Trial 2.

Plantlets were received on 7 May and were removed from culture tubes and washed free of agar on 8 to 9 May. The largest 36 plantlets of each of the five independently transformed plant lines (CYS 11, CYS 25, CYS 55, CYS 59, and CYS 71) and non-transformed (NTr) plantlets were planted into 12 perforated plastic crates (55.9 X 35.6 X 15.2 cm) filled to 10.2 cm deep with soil taken from either a nontreated (NT) area, or with soil from a preplant fumigated area (PP) (1,3-dichlororpropene [Telone II, Dow AgroSciences, Indianapolis, IN] at 374.2 l ha-1 plus metam-sodium [Amvac, Los Angeles, CA] at 702 l ha-) that had been treated on 20 July 2013. Each crate contained 18 plantlets in 6 rows (**Figure 1C**). Each row consisted of 3 plantlets from one plant line marked with colored stakes, and the rows within each crate were randomized. Plantlets were planted in the crates on 9 May. From 9 to 22 May, the crates were kept indoors under 24-hour lights. On 23 May, the crates were placed in a furrow in the field (**Figure 1B**) and later surrounded with soil. Crates were placed in the same field soil treatment as the soil in the crate. An additional crate was placed upside down to create a protective cover. On 27 May, half of the crates were treated with an organophosphate (fosthiazate 10G [Nemathorin, Syngenta International AG, Basel, Switzerland] at 4.5 kgha-1). Thus, 16 plantlets of each line were exposed to four different soil treatments in a randomized complete block design. On 25 July 2014, the foliage quality was visually rated. All visual ratings in all trials were conducted subjectively on a scale from 1-10 with 10 being the best by comparison with other plants in the same trial by an observer with more than 30 years of experience at conducting visual ratings of Easter lilies.

Prior to harvest, on 13 September 2014, the plants were again rated visually using the same scale. The number of surviving plants was determined. Plant weight and root weight were determined, and lesion nematodes were counted after removal from roots using a modified Baermann funnel technique (Ayoub, 1977). A plastic mesh screen was suspended in a cup of water, roots were placed on the screen, submerged in water, and covered with a plastic lid. Nematodes were allowed to emerge for 48 hours at which time, those that emerged into the water were counted using a stereoscopic microscope.

The harvested plants were destroyed. On 9 May, the remaining smaller plantlets to be used in Trial 2 were planted in plastic crates filled with Pro-mix HP potting mix and grown in the greenhouse until 4 November 2014.

### 2.2 Trial 2: November 2014 to September 2015

This field release was covered under the APHIS permit 15-036-102n. The plants that had been growing in the greenhouse from 9 May to 4 November 2014 were harvested and the number were counted and visually sorted into large, medium and small size categories for each line. The total number available for each line were: 96 non-transformed NW, 60 CYS 71, 36 CYS 11, 108 CYS 55, 84 CYS 59, and 72 CYS 25.

These were divided among 72 plastic mesh bags similar to those used for marketing produce (**Figure 1D**) from which they would be planted in the field to become 3 replicates in each of 4 soil treatments per line (3 replicates X 4 soil treatments X 6 lines = 72). The four soil treatments were: 1) NT (nontreated), 2) PP (fumigation on July 25, 2014 as in the previous trial), 3) AP (an at planting treatment of phorate [Thimet, Amvac, Los Angeles, CA] at 24.4 kgha-1 plus Ethoprop [Mocap, Amvac, Los Angeles, CA] at 20.9 lha-1, and 4) PP/AP (treatments 2 and 3 combined).

Prior to planting, bags were dipped for one hour at 12°C in a freshly made fungicide solution of 0.72 kg a.i. pentachloronitrobenzene (Terraclor 400, PCNB, 40% pentachloronitrobenzene, Uniroyal Chemical Company, Middlebury, CT), 0.95 kg a.i. tetramethylthiuram disulfide (42-S Thiram, 42% tetramethylthiuram disulfide, Gustafson, Plano, TX), 0.11 kg a.i. Thiophante-methyl (Systec FL 46.2%, Regal Chemical Company, Alpharetta, GA) and 0.81 kg a.i. carboxin (Vitavax-34, Gustafson, Plano, TX) per 379 liters of water and planted 9 November, 2014, within 24 hours of treatment.

**Figure 2:**
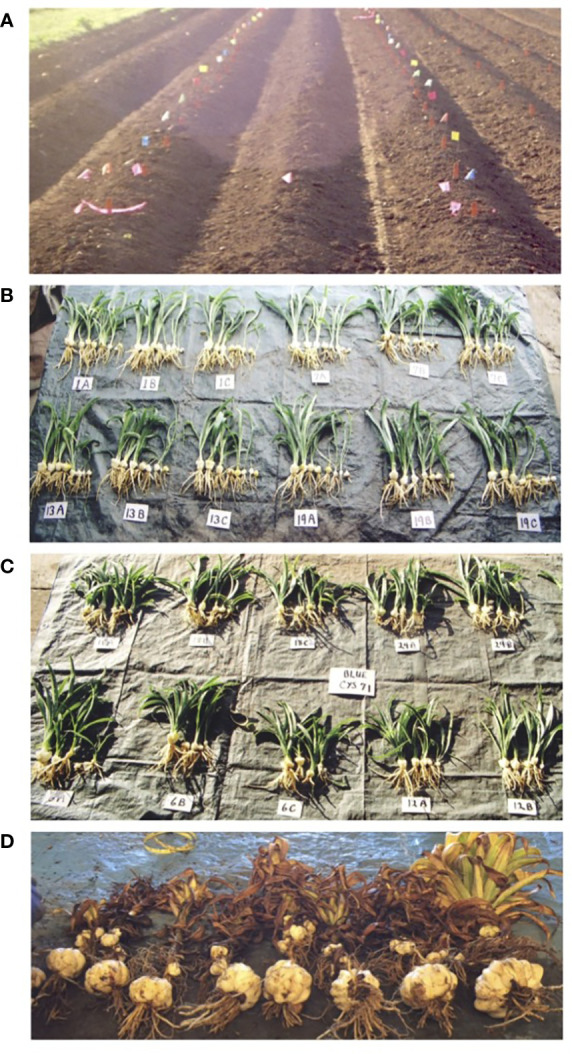
**(A)** View of field plot following planting of Trial 2. The location of each treatment was marked with a colored ribbon. **(B)** View of three replicates (a–c) of four treatments harvested from the Non-transformed Easter lily treatments in Trial 2 ready to be planted in Trial 3. Treatment 1 - Nontransformed Nellie White (NTrNW) planted in preplant fumigated soil followed by an at plant organophosphate (PP/AP), Treatment 7 - Nontransformed Nellie White (NTrNW) planted in preplant fumigated soil (PP), Treatment 13 - Nontransformed Nellie White (NTrNW) planted in nontreated soil followed by an organophoshate application (AP), Treatment 19 - Nontransformed Nellie White (NTrNW) planted in nontreated soil (NT). **(C)** View of three replicates (a–c) of four treatments of transformed lily CYS 71 harvested in Trial 2 ready to be planted in Trial 3. Treatment 18 - CYS 71 that had been planted in nontreated soil (NT) followed by at plant organophosphate (AP), Treatment 24 - CYS 71 that had been planted in nontreated soil (NT), Treatment 6 - CYS 71 that had been planted in soil treated preplant with a fumigant followed by an at plant treatment with an organophosphate (PP/AP), Treatment 12 - CYS 71 planted in soil treated preplant with a fumigant (PP). **(D)** A representative replicate of transgenic lilies harvested from Trial 3 that had been planted with bulbs harvested from Trial 2 that are pictured in **(C)**.

Plants were hand planted and harvested. Plots were 1.5 m long with 1.2 m between plots (**Figure 2A**). The numbers of plants per plot ranged from 3 to 9 depending on the line. The trial area was separated from other lilies by at least one row. Emergence was determined on 10 February, 16 February, and 27 March 2015. Visual ratings were conducted mid-season on 3 June and on 18 September 2015. Plants were hand dug, washed, and graded on 21 September 2015. If fewer than five plants were harvested from a replicate, all plants were evaluated. If five or more plants were harvested, the largest, the smallest, and three randomly selected intermediate plants were evaluated. Data collected at harvest included survival, bulb weight, visual rating of basal roots and stem roots (1 to 10 with 10 being the highest score), stem weight, and number and weight of bulblets that grew on the stems. Basal roots were removed from the bulbs, weighed, and then placed in a modified Baermann apparatus (Ayoub, 1977) for nematode extraction for two days at which time the number of nematodes were counted. Bulbs from this trial were saved for planting the subsequent year (**Figures 2B, C**).

The entire yield of each replicate harvested (with the exception of CYS 11) was placed into a separate plastic mesh bag from which to be planted in Trial 3. CYS 11 bulbs were not saved due to poor growth and health in Trials 1 and 2.

### 2.3 Trial 3: October 2015 to September 2016

This field release was done under the APHIS permit 16-033-103n. The individual mesh bags from Trial 2 were fungicide dipped on 14 October 2015 as in the previous trial. The bulbs were planted 15 October in a different location at the ELRF Research Station using the same plot design and soil treatments as the previous year. Plots were 1.5 m long with 1.2 m between plots. PP fumigation had been conducted 16 July 2015 and the AP treatment was applied at planting. A visual rating was conducted on 1 June 2016. The trial was harvested on 21 September 2016 (**Figure 2D**). Plants were harvested by shovel and hand-washed. If fewer than 5 plants were harvested from a replicate, all plants were evaluated. If 5 or more plants were harvested, the largest, the smallest, and 3 randomly selected intermediate plants were evaluated. Survival was determined, and the circumference of the bulbs was measured using calipers. Circumference was converted to grams using a previously developed regression formula: grams = 7.542417 × centimeters − 62.54368 (Westerdahl et al., 2003). Basal and stem roots were visually rated as before. Basal roots were removed and weighed followed by nematode extraction as before. Bulblets were removed from the stems, counted, and weighed. Following bulblet removal, the stems were rated visually and weighed.

Data was analyzed by Analysis of Variance (ANOVA) (*P ≤ 0.05*) followed by Fisher’s Protected Least Significant Difference Test to facilitate comparison of 1) all treatments with each other and in particular with NT/NTr NW, and 2) treatments within each of the four soil treatment groups (PP, PP/AP, AP, and NT) compared to NTr NW in that same group (JMP Pro 16, SAS Institute, Cary NC).

## 3 Results and discussion

Data has been summarized and statistically analyzed to facilitate comparisons of all treatments with each other, and comparison of treatments within a soil treatment group (**Tables 1**–**6**). Results are summarized here for 1) all treatments compared to nontreated (NT) non-transformed (NTr) Nellie White (NW) and 2) comparison within the four soil treatment groups (PP, PP/AP, AP, NT) for transformed lines compared to NTrNW.

**Table 1:** Densities of lesion nematode (*Pratylenchus penetrans*) per gram of roots at harvest in three field trials conducted in naturally infested soil at the Easter Lily Research Foundation Research Station in Brookings, OR, USA.

Soil Treatment	Lily	Trial 1		Trial 2		Trial 3	
PP/AP	NTrNW	0.09	b,x	0.04	c,y	4.43	bcd,x
PP/AP	CYS 11	0.00	b,y	0.73	bc,x	not tested	
PP/AP	CYS 25	0.00	b,y	0.19	c,xy	0.74	efg,yz
PP/AP	CYS 55	0.04	b,xy	0.17	c,xy	0.42	g,z
PP/AP	CYS 59	0.00	b,y	0.05	vc,y	2.50	bcdefg,y
PP/AP	CYS 71	0.00	b,y	0.05	c,y	0.75	efg,yz
PP	NTrNW	0.52	a,x	0.02	c,x	5.45	ab,x
PP	CYS 11	0.00	b,x	0.80	bc,x	not tested	
PP	CYS 25	0.00	b,x	0.05	c,x	1.43	defg,y
PP	CYS 55	0.00	b,x	0.29	c,x	2.14	cdefg,y
PP	CYS 59	0.00	b,x	0.07	c,x	1.28	defg,y
PP	CYS 71	0.00	b,x	0.59	c,x	0.68	fg,y
AP	NTrNW	0.14	ab,x	1.33	abc,xy	8.54	ab,x
AP	CYS 11	0.00	b,x	5.13	a,x	not tested	
AP	CYS 25	0.00	b,x	1.03	bc,y	1.25	defg,y
AP	CYS 55	0.22	ab,x	1.93	abc,xy	5.11	bc,xy
AP	CYS 59	0.00	b,x	2.51	abc,xy	1.49	defg,y
AP	CYS 71	0.00	b,x	1.59	abc,xy	3.70	bcdefg,y
NT	NTrNW	0.49	a,x	0.49	c,x	0.45	g,x
NT	CYS 11	0.00	b,y	4.62	abc,x	not tested	
NT	CYS 25	0.00	b,y	0.46	c,x	0.82	efg,x
NT	CYS 55	0.00	b,y	1.74	abc,x	4.03	bcde,x
NT	CYS 59	0.00	b,y	5.16	a,x	0.43	g,x
NT	CYS 71	0.00	b,y	1.82	abc,x	3.78	bcdef,x

Each figure is the mean of three replicates. Means followed by the same letter are not significantly different at P ≤ 0.05 either comparing all treatments in a trial (ab), or within a soil treatment group (xy). Trials 1 and 2 were planted with tissue cultured plantlets. Each replicate in Trial 3 was planted with surviving bulbs from the same treatment replicate in Trial 2. Trials tested the effects of four soil treatments: preplant fumigant (PP), after planting or at plant organophosphate (AP), a combination of preplant fumigation followed by after planting or at plant organophosphate (PP/AP), or nontreated (NT) soil that were planted to six different lines of Easter lily: non-transformed Nellie White variety (NTrNW) and five transformed lines of Nellie White (CYS 11 CYS 25, CYS 55, CYS 59, CYS 71) except that CYS 11 was not tested in Trial 3.

**Table 2:** Size of transformed and non-transformed plants (Trial 1) and bulbs at harvest (Trials 2 and 3) in field trials conducted at the Easter Lily Research Foundation Research Station in Brookings, OR, USA in soil naturally infested with lesion nematode (*Pratylenchus penetrans*).

Soil treatment	Lily	Trial 1		Trial 2		Trial 3	
		Weight (grams)		Weight (grams)		Weight (grams)	
PP/AP	NTrNW	18.54	abc,x	47.41	ab,xy	105.73	abc,xy
PP/AP	CYS 11	4.03	c,x	26.77	cdefgh,z	not tested	
PP/AP	CYS 25	21.09	abc,x	33.57	bcdef,yz	82.10	cdefg,z
PP/AP	CYS 55	19.76	abc,x	51.79	a,xy	114.03	a,xy
PP/AP	CYS 59	19.90	abc,x	43.06	abc,xyz	88.36	bcdefg,yz
PP/AP	CYS 71	15.48	abc,x	54.45	a,x	99.34	abcde,xyz
PP	NTrNW	22.42	abc,x	32.96	bcdef,x	94.23	abcdefg,xy
PP	CYS 11	33.05	ab,x	10.93	h,y	not tested	
PP	CYS 25	19.18	abc,x	23.54	efgh,xy	70.28	g,y
PP	CYS 55	15.04	abc,x	32.09	bcdefgh,x	99.02	abcde,x
PP	CYS 59	36.90	a,x	26.44	defgh,xy	96.15	abcdef,xy
PP	CYS 71	12.71	abc,x	30.01	cdefg,x	75.39	efg,xy
AP	NTrNW	14.52	abc,x	39.96	abcde,x	113.07	ab,x
AP	CYS 11	1.84	c,z	10.74	h,z	not tested	
AP	CYS 25	10.18	bc,xyz	29.54	cdefg,xy	96.47	abcdef,x
AP	CYS 55	5.03	c,xyz	28.27	cdefg,xy	102.53	abcd,x
AP	CYS 59	12.18	abc,xy	20.85	fgh,yz	100.87	abcd,x
AP	CYS 71	3.85	c,yz	27.64	cdefg,xy	102.21	abcd,x
NT	NTrNW	10.74	bc,xy	19.71	fgh,y	80.82	defg,x
NT	CYS 11	2.27	c,y	29.62	cdefg,xy	not tested	
NT	CYS 25	12.54	abc,x	15.61	gh,y	73.67	fg,x
NT	CYS 55	7.47	c,xy	26.61	cdefgh,xy	91.68	abcdefg,x
NT	CYS 59	4.66	c,xy	40.94	abcd,x	83.12	cdefg,x
NT	CYS 71	2.01	c,y	23.80	efgh,xy	101.00	abcd,x

Each figure is the mean of three replicates. Data was analyzed by Analysis of Variance (ANOVA) followed by Fisher’s Protected Least Significant Difference Test. Means followed by the same letter are not significantly different at P ≤ 0.05 either comparing all treatments in a trial (ab), or within a soil treatment group (xy). Trials 1 and 2 were planted with tissue cultured plantlets. Each replicate in Trial 3 was planted with surviving bulbs from the same treatment replicate in Trial 2. Trials tested the effects of four soil treatments: preplant fumigant (PP), after planting or at plant organophosphate (AP), a combination of preplant fumigation followed by after planting or at plant organophosphate (PP/AP), or nontreated (NT) soil that were planted to six different lines of Easter lily: non-transformed Nellie White variety (NTrNW) and five transformed lines of Nellie White (CYS 11 CYS 25, CYS 55, CYS 59, CYS 71) except that CYS 11 was not tested in Trial 3.

**Table 3:** Size of largest and smallest bulbs at harvest in field trials conducted at the Easter Lily Research Foundation Research Station in Brookings, OR, USA in soil naturally infested with lesion nematode (*Pratylenchus penetrans*).

Soil treatment	Lily	Largest Bulb				Smallest Bulb			
		Trial 2 (grams)		Trial 3 (grams)		Trial 2 (grams)		Trial 3 (grams)	
PP/AP	NTrNW	99.11	bcde,xy	143.40	bcde,y	12.10	bcde,xy	39.63	abcd,xy
PP/AP	CYS 11	35.81	hijk,z	not tested		17.73	abcd,x	not tested	
PP/AP	CYS 25	94.04	bcde,yz	130.63	cde,y	2.78	e,y	31.65	abcd,xy
PP/AP	CYS 55	156.00	a,x	168.95	ab,x	7.40	cde,y	49.21	abc,x
PP/AP	CYS 59	105.19	bcde,xy	137.02	bcde,y	10.28	bcde,xy	8.50	d,y
PP/AP	CYS 71	114.60	abc,xy	159.37	abc,x	19.40	abcd,x	34.84	abcd,xy
PP	NTrNW	121.86	abc,x	156.17	abcd,x	5.42	de,x	46.02	abc,x
PP	CYS 11	20.65	jk,z	not tested		3.08	e,x	not tested	
PP	CYS 25	67.20	defghi,yz	146.59	bcde,xy	4.57	e,x	26.86	bcd,x
PP	CYS 55	83.93	bcdef,xy	146.59	bcde,xy	6.36	de,x	36.44	abcd,x
PP	CYS 59	81.50	bcdefg,xy	143.40	bcde,xy	3.06	e,x	46.02	abc,x
PP	CYS 71	47.54	fghijk,yz	132.23	cde,y	8.50	cde,x	30.05	abcd,x
AP	NTrNW	83.20	bcdefg,x	162.56	abc,xy	8.01	cde,x	52.40	abc,x
AP	CYS 11	13.60	k,y	not tested		7.87	cde,x	not tested	
AP	CYS 25	94.92	bcde,x	183.31	a,x	3.73	e,x	23.67	cd,x
AP	CYS 55	63.31	defghij,x	140.21	bcde,y	8.04	cde,x	49.21	abc,x
AP	CYS 59	63.56	defghij,x	141.81	bcde,y	1.86	e,x	58.79	ab,x
AP	CYS 71	55.04	efghijk,xy	154.58	abcd,xy	13.08	bcde,x	52.40	abc,x
NT	NTrNW	44.32	fghijk,xy	119.45	ef,x	4.09	e,y	52.40	abc,x
NT	CYS 11	32.96	ijk,y	not tested		26.27	a,x	not tested	
NT	CYS 25	38.44	ghijk,y	97.10	f,x	2.68	e,y	50.81	abc,x
NT	CYS 55	80.46	bcdefgh,x	137.02	bcde,x	5.71	de,y	33.25	abcd,x
NT	CYS 59	72.20	cdefghi,xy	141.81	bcde,x	20.87	ab,xy	25.26	cd,x
NT	CYS 71	44.55	fghijk,xy	125.84	def,x	8.16	cde,xy	60.39	a,x

Each figure is the mean of three replicates. Data was analyzed by Analysis of Variance (ANOVA) followed by Fisher’s Protected Least Significant Difference Test. Means followed by the same letter are not significantly different at P ≤ 0.05 either comparing all treatments in a trial (ab), or within a soil treatment group (xy). Trial 2 was planted with tissue cultured plantlets. Each replicate in Trial 3 was planted with surviving bulbs from the same treatment replicate in Trial 2. Trials tested the effects of four soil treatments: preplant fumigant (PP), after planting or at plant organophosphate (AP), a combination of preplant fumigation followed by after planting or at plant organophosphate (PP/AP), or nontreated (NT) soil that were planted to six different lines of Easter lily: non-transformed Nellie White variety (NTrNW) and five transformed lines of Nellie White (CYS 11 CYS 25, CYS 55, CYS 59, CYS 71) except that CYS 11 was not tested in Trial 3.

**Table 4:** Weight of basal roots (grams) per plant at harvest in field trials conducted at the Easter Lily Research Foundation Research Station in Brookings, OR, USA in soil naturally infested with lesion nematode (*Pratylenchus penetrans*).

Soil treatment	Lily	Trial 1		Trial 2		Trial 3	
PP/AP	NTrNW	5.25	ab,x	16.36	bcde,w	10.40	a,x
PP/AP	CYS 11	1.75	bcde,x	6.45	ghi,x	not tested	
PP/AP	CYS 25	4.65	abcd,x	19.94	abc,w	7.87	abcde,x
PP/AP	CYS 55	4.43	abcde,x	13.08	cdefg,wx	9.33	abc,x
PP/AP	CYS 59	4.79	abc,x	20.09	abc,w	8.13	abcd,x
PP/AP	CYS 71	2.77	abcde,x	15.96	bcde,w	7.73	abcde,x
PP	NTrNW	4.10	abcde,x	6.66	fghi,yz	8.13	abcd,x
PP	CYS 11	1.82	bcde,x	3.91	hi,z	not tested	
PP	CYS 25	5.33	ab,x	21.54	ab,w	6.67	bcdefg,x
PP	CYS 55	3.47	abcde,x	14.54	bcdef,wxy	6.27	cdefg,x
PP	CYS 59	6.63	a,x	17.16	bcd,wx	7.33	abcdef,x
PP	CYS 71	3.10	abcde,x	8.55	efghi,xyz	4.13	fg,x
AP	NTrNW	5.26	ab,x	4.39	hi,z	9.20	abc,x
AP	CYS 11	0.44	e,z	1.22	i,z	not tested	
AP	CYS 25	2.88	abcde,xyz	18.04	bcd,w	9.96	ab,x
AP	CYS 55	1.89	bcde,yz	12.42	cdefg,x	7.47	abcdef,x
AP	CYS 59	3.99	abcde,xyz	11.17	defgh,x	8.96	abcd,x
AP	CYS 71	0.75	de,z	2.01	i,z	4.40	efg,y
NT	NTrNW	3.20	abcde,xy	3.59	hi,z	6.67	bcdefg,x
NT	CYS 11	1.18	cde,xy	5.22	ghi,z	not tested	
NT	CYS 25	3.70	abcde,x	16.62	bcd,x	5.49	defg,x
NT	CYS 55	1.96	bcde,xy	12.41	cdefg,xy	5.60	defg,x
NT	CYS 59	2.54	bcde,xy	26.91	a,w	6.62	bcdefg,x
NT	CYS 71	0.46	e,y	2.73	i,y	3.50	g,x

Each figure is the mean of three replicates. Data was analyzed by Analysis of Variance (ANOVA) followed by Fisher’s Protected Least Significant Difference Test. Means followed by the same letter are not significantly different at P ≤ 0.05 either comparing all treatments in a trial (ab), or within a soil treatment group (xy). Trials 1 and 2 were planted with tissue cultured plantlets. Each replicate in Trial 3 was planted with surviving bulbs from the same treatment replicate in Trial 2. Trials tested the effects of four soil treatments: preplant fumigant (PP), after planting or at plant organophosphate (AP), a combination of preplant fumigation followed by after planting or at plant organophosphate (PP/AP), or nontreated (NT) soil that were planted to six different lines of Easter lily: non-transformed Nellie White variety (NTrNW) and five transformed lines of Nellie White (CYS 11 CYS 25, CYS 55, CYS 59, CYS 71) except that CYS 11 was not tested in Trial 3. Visual ratings in all trials were conducted subjectively on a scale from 1-10 with 10 being the best by comparison with other plants in the same trial by an observer with more than 30 years of experience at conducting visual ratings of Easter lilies.

**Table 5:** Basal root visual rating at harvest in field trials conducted at the Easter Lily Research Foundation Research Station in Brookings, OR, USA in soil naturally infested with lesion nematode (*Pratylenchus penetrans*).

Soil treatment	Lily	Trial 1		Trial 2		Trial 3	
PP/AP	NTrNW	7.67	a,x	8.16	a,x	9.67	a,x
PP/AP	CYS 11	4.67	a,x	8.17	a,x	not tested	
PP/AP	CYS 25	8.00	a,x	7.46	ab,x	8.67	abc,xy
PP/AP	CYS 55	4.67	a,x	7.96	a,x	7.67	abcd,xy
PP/AP	CYS 59	6.33	a,x	7.59	ab,x	8.33	abc,xy
PP/AP	CYS 71	4.67	a,x	7.09	abc,x	6.67	cd,y
PP	NTrNW	7.00	a,x	6.61	abcd,x	9.67	a,x
PP	CYS 11	5.33	a,x	5.39	bcdefg,x	not tested	
PP	CYS 25	8.33	a,x	5.80	abcdef,x	8.00	abcd,xy
PP	CYS 55	9.00	a,x	6.29	abcde,x	7.67	abcd,yz
PP	CYS 59	7.33	a,x	6.64	abcd,x	8.33	abc,xy
PP	CYS 71	4.67	a,x	6.85	abcd,x	6.00	d,z
AP	NTrNW	7.33	a,x	4.89	cdefgh,x	9.67	a,x
AP	CYS 11	4.33	a,x	2.33	i,y	not tested	
AP	CYS 25	7.33	a,x	4.93	cdefgh,x	9.00	ab,xy
AP	CYS 55	5.00	a,x	4.12	efghi,xy	8.67	abc,xy
AP	CYS 59	7.67	a,x	2.68	hi,xy	9.00	ab,xy
AP	CYS 71	4.67	a,x	3.73	fghi,xy	7.33	bcd,y
NT	NTrNW	8.00	a,x	4.51	defghi,x	8.33	abc,x
NT	CYS 11	4.67	a,x	3.67	fghi,x	not tested	
NT	CYS 25	6.33	a,x	4.42	defghi,x	8.00	abcd,x
NT	CYS 55	7.33	a,x	4.47	defghi,x	7.67	abcd,x
NT	CYS 59	5.67	a,x	3.69	fghi,x	7.33	bcd,x
NT	CYS 71	4.00	a,x	2.98	ghi,x	7.00	bcd,x

Each figure is the mean of three replicates. Data was analyzed by Analysis of Variance (ANOVA) followed by Fisher’s Protected Least Significant Difference Test. Means followed by the same letter are not significantly different at P ≤ 0.05 either comparing all treatments in a trial (ab), or within a soil treatment group (xy). Trials 1 and 2 were planted with tissue cultured plantlets. Each replicate in Trial 3 was planted with surviving bulbs from the same treatment replicate in Trial 2. Trials tested the effects of four soil treatments: preplant fumigant (PP), after planting or at plant organophosphate (AP), a combination of preplant fumigation followed by after planting or at plant organophosphate (PP/AP), or nontreated (NT) soil that were planted to six different lines of Easter lily: non-transformed Nellie White variety (NTrNW) and five transformed lines of Nellie White (CYS 11 CYS 25, CYS 55, CYS 59, CYS 71) except that CYS 11 was not tested in Trial 3.

**Table 6:** Percent survival of Easter lily plants at harvest in field trials conducted at the Easter Lily Research Foundation Research Station in Brookings, OR, USA in soil naturally infested with lesion nematode (*Pratylenchus penetrans*).

Soil Treatment	Lily	Trial 1		Trial 2		Trial 3	
PP/AP	NTrNW	88.89	ab,xy	91.67	a,x	87.50	abc,x
PP/AP	CYS 11	46.67	b,y	55.56	bc,y	not tested	
PP/AP	CYS 25	88.89	ab,xy	88.89	a,x	100.00	a,x
PP/AP	CYS 55	88.89	ab,xy	85.19	ab,x	100.00	a,x
PP/AP	CYS 59	100.00	a,x	85.71	a,x	100.00	a,x
PP/AP	CYS 71	66.67	ab,xy	86.67	a,x	100.00	a,x
PP	NTrNW	77.78	ab,x	87.50	a,xy	95.83	a,x
PP	CYS 11	66.67	ab,x	100.00	a,x	not tested	
PP	CYS 25	100.00	a,x	72.22	abc,y	88.89	abc,x
PP	CYS 55	77.78	ab,x	81.48	ab,xy	88.89	abc,x
PP	CYS 59	77.78	ab,x	85.71	a,xy	100.00	a,x
PP	CYS 71	66.67	ab,x	93.33	a,xy	100.00	a,x
AP	NTrNW	88.89	ab,x	83.33	ab,x	95.83	a,x
AP	CYS 11	55.56	ab,x	55.56	bc,x	not tested	
AP	CYS 25	77.78	ab,x	83.33	ab,x	83.33	abcd,x
AP	CYS 55	44.44	b,x	74.08	abc,x	81.48	abcd,x
AP	CYS 59	66.67	ab,x	80.95	ab,x	57.14	cd,x
AP	CYS 71	66.67	ab,x	80.00	ab,x	80.00	abcd,x
NT	NTrNW	77.78	ab,x	83.33	ab,x	83.33	abcd,x
NT	CYS 11	44.44	b,x	44.44	c,x	not tested	
NT	CYS 25	77.78	ab,x	72.22	abc,x	61.11	cd,x
NT	CYS 55	55.56	ab,x	74.08	abc,x	92.59	ab,x
NT	CYS 59	66.67	ab,x	47.62	c,x	66.67	bcd,x
NT	CYS 71	66.67	ab,x	73.33	abc,x	60.00	cd,x

Each figure is the mean of three replicates. Data was analyzed by Analysis of Variance (ANOVA) followed by Fisher’s Protected Least Significant Difference Test. Means followed by the same letter are not significantly different at P ≤ 0.05 either comparing all treatments in a trial (ab), or within a soil treatment group (xy). Trials 1 and 2 were planted with tissue cultured plantlets. Each replicate in Trial 3 was planted with surviving bulbs from the same treatment replicate in Trial 2. Trials tested the effects of four soil treatments: preplant fumigant (PP), after planting or at plant organophosphate (AP), a combination of preplant fumigation followed by after planting or at plant organophosphate (PP/AP), or nontreated (NT) soil that were planted to six different lines of Easter lily: non-transformed Nellie White variety (NTrNW) and five transformed lines of Nellie White (CYS 11 CYS 25, CYS 55, CYS 59, CYS 71) except that CYS 11 was not tested in Trial 3.

The transformed lines were not totally resistant to lesion nematode, but significant nematode reductions were documented with and without chemical soil treatments (**Table 1**). This was most evident in Trial 1 where 19 of 20 transformed lines grown in various soil treatments had lower levels of lesion nematode than NT NTrNW (*P ≤ 0.05)* and in Trial 3 where nematode levels in all transformed lines were lower than corresponding NTrNW for PP/AP, PP, and AP soil treatments (*P ≤ 0.05)*. Determining degree of nematode control is confounded by root growth (**Table 4**) and health (**Table 5**). Roots that have been damaged by nematode or fungal infestation may not be able to support nematode levels as high as can healthy roots. For example, in Trial 3, the relatively small size of root systems in NT NTrNW (**Table 4**) compared to PP/AP NTrNW could be related to lower levels of lesion nematode in NT NTrNW than in PP/AP NTrNW.

When planted in Trial 2, the plantlets were not infested. There was potential for the roots to become infested during the trial and for this infestation to be carried over into Trial 3. This is a normal progression in the culture of Easter lilies. Bulblets planted commercially are potentially infested with low levels of lesion nematode and are planted into nematode infested soil.

Comparing all treatments in Trial 1, lesion nematode was numerically lower than NT NTrNW for all PP/AP; all PP except NTrNW; for all AP except NTrNW and CYS 55 and for NT CYS 71 (**Table 1**). Comparing all treatments to NT NTrNW, at *P ≤ 0.05*, lesion nematode was lower for all PP/AP; all PP except NTrNW; all AP except NTrNW and CYS 55; and for all NT. Within soil treatment groups at *P ≤ 0.05* lesion nematode was lower than NTrNW for all PP/AP except CYS 55; and all NT.

Comparing all treatments in Trial 2, numerically lesion nematode was lower than NT NTrNW for all PP/AP except CYS 11; PP NTrNW, CYS 25, CYS 55, and CYS 59; and NT CYS 25. Comparing all treatments to NT NTrNW, at *P ≤ 0.05*, lesion nematode was higher in AP CYS 11; and NT CYS 11 and CYS 59. Within soil treatment groups at *P ≤ 0.05* lesion nematode was greater than NTrNW in PP/AP CYS 11

Comparing all treatments in Trial 3, numerically, only PP/AP CYS 55 and NT CYS 59 were lower than NT NTrNW. Numerically within soil treatment groups, lesion nematode was lower than NTrNW for all PP/AP, PP, and AP treatments. The same is true at *P ≤ 0.05* except for AP CYS 55.

The size and appearance of bulbs is the primary criterion of marketability of bulbs to greenhouses for forcing. Seasonal weather patterns greatly affect quality and size of bulbs even in the absence of nematode pests. For example, NW bulbs produced one year can be more than double the size of those produced in another year (Roberts et al., 1985).

Trials conducted at the ELRF Station rotate through four different fields. Therefore, in addition to weather variation, there is additional variability in soil characteristics and nematode population levels. Even the standard products utilized by growers have shown year-to-year variability working better in some years than others (L.J. Riddle, pers. comm.). The PP and AP chemical treatments used in these trials have been developed over many years of research and always provide superior growth compared to nontreated soil. However, there are years when the PP or AP treatments alone provide better growth than the combined PP/AP treatment. Comparing the size of NW bulbs, in all three trials, the PP/AP, PP, and AP soil treatments all resulted in better growth than NT. PP provided the best growth in Trial 1, PP/AP in Trial 2, and AP in Trial 3 (**Table 2**).

Easter lily bulbs are typically calipered, boxed and sold based on circumference with larger bulbs being sold at a higher price. Bulbs were weighed in Trials 1 and 2 because they were too small to caliper for circumference measurements. For comparison with Trials 1 and 2, the circumference data for Trial 3 was converted to grams using a previously developed regression formula: grams = 7.542417 × centimeters − 62.54368 (Westerdahl et al., 2003).

Even though Trial 1 was in the ground for less than half a normal growing season, many of the transformed lines were numerically larger at harvest than NT NTrNW and were equivalent to NTrNW within soil treatment groups (*P ≤ 0.05)* (**Table 2**). In trial 2, with the exception of CYS 11, most transformed lines were numerically larger than NT NTrNW and those in the PP/AP soil treatment groups CYS 55. CYS 59, and CYS 71 were significantly larger (*P ≤ 0.05).* Because of poor vigor, CYS 11 was eliminated from consideration after Trial 2. In Trial 3, growth of the transformed lines was essentially equivalent to that of NTrNW demonstrating that transformed lilies retain their vigor for at least two years. Looking at the largest bulb that developed in Trial 2, with the exception of CYS 11, compared to NT NTrNW, the largest bulbs developed in the PP/AP soil treatment group (*P ≤ 0.05*) (**Table 3**). In Trial 3, growth of the transformed lines was for the most part equivalent to that of NTrNW. In Trials 2 and 3, growth of the smallest bulbs harvested was typically equivalent to NTrNW (**Table 3**).

In Trial 1, numerically, over all treatments plant weight was greater than NT NTrNW for all PP/AP except CYS 11; all PP; AP NTrNW and CYS 59; and NT CYS 25. Comparing all treatments to NT NTrNW, at *P ≤ 0.05*, plant weight was greater for PP CYS 59. Within soil treatment groups at *P ≤ 0.05* plant weight was equivalent to NTrNW with the exception of AP CYS 11.

In Trial 2, numerically, over all treatments. average bulb weight was greater than NT NTrNW for all PP/AP; all PP except CYS 11; all AP except CYS 11; and all NT except CYS 25. Over all treatments compared to NT NTrNW, at *P ≤ 0.05* average bulb weight was greater for PP/AP NTrNW, CYS 55, CYS 59 and CYS 71; AP NTrNW; and NT CYS 59. Within soil treatment groups at *P ≤ 0.05* average bulb weight was less than NTrNW for PP/AP CYS 11; PP CYS 11; AP CYS 11 and CYS 59; and greater for NT CYS 59.

In Trial 3, numerically, over all treatments, bulb circumference was greater than NT NTrNW for all treatments except PP CYS 25 and CYS 71. Over all treatments compared to NT NTrNW, at *P ≤ 0.05* bulb circumference was greater for PP/AP NTrNW and CYS 55; and AP NTrNW. Within soil treatments groups, there were no differences at *P ≤ 0.05* except that PP/AP CYS 25 bulbs were smaller than NTrNW.

In Trial 2, numerically, over all treatments the weight of the largest bulb was greater than NT NTrNW for all PP/AP except CYS 11; all PP except CYS 11; all AP except CYS 11; and NT CYS 11, and CYS 25 (**Table 3**). Over all treatments compared to NT NTrNW, at *P ≤ 0.05* the weight of the largest bulb was greater for all PP/AP except CYS 11; PP NTrNW; and AP CYS 25. Within soil treatment groups at *P ≤ 0.05* the weight of the largest bulb was less than NTrNW in that group for PP/AP CYS 11; PP CYS 11, CYS 25 and CYS71; and for AP CYS 11.

In Trial 3, numerically, over all treatments the weight of the largest bulb was larger than NT NTrNW for all treatments except NT CYS 25. Over all treatments compared to NT NTrNW, at *P ≤ 0.05* the weight of the largest bulb was larger for PP/AP CYS 55 and CYS 71; PP CYS 71; and for AP NTrNW, CYS 25 and CYS 75. Within soil treatment groups at *P ≤ 0.05* the weight of the largest bulb was larger than NTrNW for PP/AP CYS 55 and CYS 71; and for PP CYS 71.

In Trial 2, numerically, over all treatments the weight of the smallest bulb was greater than NT NTrNW for all PP/AP except CYS 25; all PP except CYS 11 and CYS 59; all AP except CYS 25 and CYS 59; and all NT except CYS 25. Over all treatments compared to NT NTrNW, at *P ≤ 0.05* the weight of the smallest bulb was greater for PP/AP CYS 11 and CYS 71; and for NT CYS 11 and CYS 59. Within soil treatment groups at *P ≤ 0.05* the weight of the smallest bulb was greater than NTrNW for NT CYS 11.

In Trial 3, numerically, over all treatments the weight of the smallest bulb was greater than NT NTrNW for AP CYS 59; and NT CYS 71. Over all treatments compared to NT NTrNW, at *P ≤ 0.05* the weight of the smallest bulb was less for PP/AP CYS 59. There were no significant differences within soil treatment groups.

The size and apparent health of a bulbs basal root system is also an important factor in marketability of bulbs. For the most part, root systems of transformed bulbs were of similar size to NTrNW (**Table 4**). In Trial 2, in several instances CYS 25, CYS 55, and CYS 59 had larger root systems than NTrNW (*P ≤ 0.05*). Visually, in the three trials, there were few significant differences in basal root ratings with the exception that in Trial 2, all PP/AP ratings were better than NT NTrNW (*P ≤ 0.05*) (**Table 5**).

In Trial 1, numerically, over all treatments there was a greater root weight than NT NTrNW for PP/AP NTrNW, CYS 25, CYS 55, and CYS 59; PP NTrNW, CYS 25, CYS 55, and CYS 59; AP NTrNW, and CYS 59; and NT CYS 25. Over all treatments compared to NT NTrNW, there were no significant differences (*P ≤ 0.05*). Within soil treatment groups root weight was lower for AP CYS 11, CYS 55, and CYS 71 compared to NTrNW.

In Trial 2, numerically, over all treatments, root weight was greater than NT NTrNW for all PP/AP, all PP, all AP except CYS 11, and CYS 71; and for NT all except CYS 71. Over all treatments, root weight was greater than NT NTrNW for all PP/AP except CYS 11; PP CYS 25, CYS 55, and CYS 59; AP CYS 25, and CYS 55; and for NT CYS 25, CYS 55, and CYS 59 (*P ≤ 0.05*). Within soil treatment groups root weight was greater than NTrNW for PP CYS 25, and CYS 59; AP CYS 25, CYS 55, and CYS 59; and for NT CYS 25, CYS 55 and CYS 59 (P ≤ 0.05). PP/AP CYS 11 had poorer root growth than NTrNW (*P ≤ 0.05*).

In Trial 3, numerically, over all treatments, root weight was greater than NT NTrNW for all PP/AP; PP NTrNW and CYS 59; and all AP except CYS. 71. Over all treatments, at P ≤ 0.05 only PP/AP NTrNW was better than NT NTrNW. Within soil treatment groups at P ≤ 0.05 AP CYS 71 had a lower root weight than AP NTrNW.

In Trial 1, numerically, over all treatments, PP CYS 25 and CYS 55 had a higher basal root visual score than NT NTrNW (**Table 5**). Statistically, there were no differences over all or within soil group treatments (*P ≤ 0.05*).

In Trial 2, numerically, over all treatments, the basal root visual score was greater than NT NTrNW for all PP/AP; all PP all bulbs; and AP NTrNW, and CYS 25. At *P ≤ 0.05*, overall treatments, all PP/AP were visually better than NT NTrNW. Within soil treatment groups, AP CYS 11 was not as good as NTrNW (*P ≤ 0.05*).

In Trial 3, the visual rating of basal roots showed that numerically over all treatments, PP/AP NTrNW, and CYS 25; PP NTrNW; AP NTrNW, CYS 25, CYS 55, and CYS 59 looked better than NT NTrNW. Over all treatments, PP CYS 71 had a lower visual basal root rating than NT NTrNW *(P ≤ 0.05*). Within soil treatment groups, PP/AP CYS 71, PP CYS 55 and CYS 71; and AP CYS 71 received a lower score than NTrNW in that same group (*P ≤ 0.05*).

Survival in Trial 1 ranged from 44.44 to 100 percent with lowest survival occurring in CYS 11 and CYS 55, but no statistically significant differences were delineated (*P ≤ 0.05*) (**Table 6**). Survival was again variable in Trial 2, but only significantly lower for CYS 11 and CYS 59 (*P ≤ 0.05*). There were no significant differences in survival in Trial 3 (*P ≤ 0.05*). Statistically significant differences due to variability between replicates and economically significant differences due to crop loss are not always equivalent.

Because a variable number of bulbs were planted in Trials 2 and 3, survival was analyzed on a percent basis. In Trial 1, numerically, over all treatments, survival was greater than NT NTrNW for all PP/AP except CYS 11; for PP CYS 25; and for AP NTrNW. There were no significant differences either over all or within soil treatment groups. In Trial 2, numerically, over all treatments, survival was greater than NT NTrNW for all PP/AP except CYS 11; and all PP except CYS 25 and CYS 55. Over all treatments compared to NT NTrNW, at *P ≤ 0.05* survival was lower for NT CYS 11 and CYS 59. Within soil treatment groups at *P ≤ 0.05* survival was lower than NTrNW for PP/AP CYS 11. In Trial 3, numerically, over all treatments, survival was greater than NT NTrNW for all PP/AP; all PP; AP NTrNW, and for NT CYS 55. There were no significant differences either over all or within soil treatment groups.

Time of emergence of shoots aboveground was monitored on three dates in Trial 2 (**Supplementary Material 1.1**). Statistically, there were no differences in emergence either overall or within soil treatment groups (*P ≤ 0.05*). Weather conditions prevented obtaining emergence data for Trial 3.

Plant growth and health was rated visually in all three trials (**Supplementary Material 1.2**). When rated mid-season this can provide an early indication of how plants will perform at harvest. Poor visual ratings for CYS 11 in both Trial 1 and Trial 2 (which were only significant in the AP soil group treatments) as well as there being very few surviving bulbs at harvest contributed to its not being included in Trial 3.

The weights of stems and stem root scores were highly variable (**Supplementary Material 1.3**). Stem weight and stem root visual scores are not available for Trial 1 because plants were not in the ground long enough for these characteristics to develop. Largest stem weights were associated with PP/AP soil treatment in Trial 2 and with AP soil treatment in Trial 3.

The number and size of bulblets that develop on stems belowground is important because they are often utilized in subsequent plantings (**Supplementary Material 1.4**). Trial 1 conducted May-September 2014 was planted from the tissue cultured plantlets received from USDA. This was too short a period of time to produce bulblets. Plantlets not used for Trial 1 were raised in the greenhouse until used for Trial 2. The plants in Trial 2 were in the field for a full growing season, developed bulbs at the base of the plant that were to plant Trial 3, and produced bulblets on the underground stems. Both number and weight of bulblets generally increased from Trial 2 to Trial 3 correlated with the greater size of bulbs. The largest number and weight of bulblets in Trial 2 were associated with the PP/AP soil treatment.

This study confirmed what others have found when comparing the resistance of plants engineered for disease and pest resistance in the field and *in vitro*. Very often plants that showed disease resistance in the greenhouse or *in vitro* were not resistant in the field. Environmental conditions differ, and the field is a much harsher environment with temperature, water, drought, salt stress, etc. A recent study found that environmental stress was the major cause of transcriptomic and proteomic changes in both GM and non-GM plants (Batista et al., 2017). The microbe environment is also complex in the field. We identified several fungi including *Fusarium oxysporum*, *F. tricinctum*, and *Rhizoctonia* sp. AG-I from necrotic roots of lilies and demonstrated *in vitro* that root lesion infection proceeds more quickly in the presence of the fungal isolates (Lakshman et al., 2017).

There have only been two field studies involving plants engineered for migratory nematode resistance, and both studies found that transgenic plants were more resistant to the infecting migratory nematodes than non-transformed plants (Gao et al., 2011; Tripathi et al., 2015). Rice plants transformed with *oryzacystatin*-1 (OC1) showed resistance to stem nematodes (*D. destructor*) in the field (Gao et al., 2011). Plantains transformed with a synthetic peptide that interferes with chemoreception or dual defense genes (a maize cystatin combined with synthetic peptide) showed resistance in the field trial in Uganda (Tripathi et al., 2015).

Development of the transgenic Easter lilies involved tissue culture techniques, and this may have affected growth characteristics of the transgenic lilies. Bulb scales of lily plants micropropagated *in vitro* were cultured four months on MS medium containing either 1 mg/L picloram (CYS 55 and CYS 59) or 2 mg/L dicamba (CYS 25) and mannitol as an osmoticum prior to bombardment with the gene gun. CYS 71 was regenerated from bombarded suspension cells of Easter lily that had been on 0.5 mg/L picloram for one year. Following bombardment, bulb scales were cultured on selection medium containing both picloram and phosphinothricin for approximately one year to select for regenerated plantlets that were putatively transformed. Somaclonal variation has been reported to occur when plants are regenerated from callus *in vitro* (Larkin and Scowcroft, 1981; Lee and Phillips, 1987; Phillips et al., 1994; Park et al., 2009). Also, some *in vitro* conditions such as growing plants on osmoticum prior to gene gun bombardment have been shown to cause cytogenetic abnormalities in transgenic barley plants (Choi et al., 2001). Fonseca et al. (2014) found proteomic differences between transgenic and non-transgenic plants that were thought to be caused *by in vitro* culture.

In our field study micropropagated, non-transformed lilies were compared to transformed lilies. It may have been informative to include lilies transformed with a vector only (lacking the cystatin gene) as an additional control so that all lilies with or without the cystatin gene had been through tissue culture. Nonetheless, the lilies with the cystatin gene had shown resistance to *P. penetrans* as compared to micropropagated lilies *in vitro* (Vieira et al., 2015). Results from our field trials demonstrate the importance of conducting field trials to determine effective resistance to nematodes.

While not totally resistant, when planted in the field, transformed lines demonstrated and maintained a degree of resistance to lesion nematode over two growing seasons and displayed desirable growth and quality characteristics similar to non-transformed lilies. They also reacted similarly to non-transformed lilies to standard soil chemical treatments. In spite of the variability that naturally occurs in field trials conducted over multiple years, our results were fairly consistent. With further development, transformed lilies could help producers meet the overall goal of reducing pesticide use.

## Data availability statement

The raw data supporting the conclusions of this article will be made available by the authors, without undue reservation.

## Author contributions

All authors listed have made a substantial, direct, and intellectual contribution to the work and approved it for publication.
