# Supplementary material for: Field test of Easter lilies transformed with a rice cystatin gene for root lesion nematode resistance
Source: Front Plant Sci. 2023 Mar 24;14:1134224. doi: 10.3389/fpls.2023.1134224 (PMC10081024; doi:10.3389/fpls.2023.1134224)
Supplement: Supplementary file 1 [file Table_1.docx]

Supplementary Material

Field test of Easter lilies transformed with a rice cystatin gene

for root lesion nematode resistance

Becky Westerdahl, Lee Riddle, Deborah Giraud, Kathryn Kamo

*** Correspondence:** Becky Westerdahl: bbwesterdahl@ucdaavis.edu

# Supplementary Tables

## Time of shoot emergence

Time of emergence of shoots aboveground was monitored on three dates in Trial 2. On the first date, emergence was lower for CYS 11 than for the other transformed lines (*P ≤ 0.05*), but there was no significant difference on the last date. The number of plants that emerged was determined on 10 February, 16 February, and 27 March 2015. Numerically, on 10 February, the percent emergence over all was greater than NT NTrNW for PP/AP NTrNW and CYS 59; PP CYS 59; and NT CYS 71. Over all treatments, at *P ≤ 0.05* emergence was lower than NT NTrNW for PP/AP CYS 11; AP CYS 11; and NT CYS 11. Within soil treatment groups there was less emergence at *P ≤ 0.05* for PP/AP CYS 11. Numerically, on 16 February, percent emergence overall was lower than NT NTrNW for all PP/AP except NTrNW; all PP except CYS 59; all AP; and all NT except CYS 71. Over all, at *P ≤ 0.05* emergence was lower for PP/AP CYS 11. Within soil treatment groups, at *P ≤ 0.05*, there was less emergence for PP/AP CYS 11. Numerically on 27 March, over all there was greater emergence than NT NTrNW for all PP/AP except CYS 11; for all PP except NTrNW and CYS 25; and for AP NTrNW and CYS 55. Statistically, there were no differences in emergence either overall or within soil treatment groups (*P ≤ 0.05*).

| Percent emergence of shoots in Trial 2. | | | | | | | | |
| --- | --- | --- | --- | --- | --- | --- | --- | --- |
| Soil treatment | Lily | 10 February | | 16 February | | | 27 March | |
| PP/AP | NTrNW | 100.00 | a,x | 100.00 | a,x | 100.00 | | a,x |
| PP/AP | CYS 11 | 44.44 | c,y | 44.44 | e,y | 77.78 | | abc,x |
| PP/AP | CYS 25 | 77.78 | abc,x | 77.78 | abcde,x | 94.44 | | ab,x |
| PP/AP | CYS 55 | 81.48 | ab,x | 81.48 | abcd,x | 92.59 | | abc,x |
| PP/AP | CYS 59 | 85.71 | ab,x | 85.71 | abcd,x | 95.24 | | ab,x |
| PP/AP | CYS 71 | 80.00 | ab,x | 80.00 | abcde,x | 93.33 | | ab,x |
| PP | NTrNW | 70.83 | abc,x | 79.17 | abcde,x | 79.17 | | abc,xy |
| PP | CYS 11 | 66.67 | abc,x | 77.78 | abcde,x | 100.00 | | a,x |
| PP | CYS 25 | 61.11 | bc,x | 61.11 | bcde,x | 61.11 | | c,y |
| PP | CYS 55 | 77.78 | abc,x | 81.48 | abcd,x | 96.30 | | ab,x |
| PP | CYS 59 | 85.71 | ab,x | 95.24 | ab,x | 95.24 | | ab,x |
| PP | CYS 71 | 80.00 | ab,x | 80.00 | abcde,x | 93.33 | | ab,x |
| AP | NTrNW | 75.00 | abc,x | 79.17 | abcde,xy | 91.67 | | abc,x |
| AP | CYS 11 | 44.45 | c,x | 55.56 | de,y | 66.67 | | bc,x |
| AP | CYS 25 | 66.67 | abc,x | 72.22 | abcde,xy | 77.78 | | abc,x |
| AP | CYS 55 | 74.08 | abc,x | 81.48 | abcd,x | 96.30 | | ab,x |
| AP | CYS 59 | 76.19 | abc,x | 76.19 | abcde,xy | 85.71 | | abc,x |
| AP | CYS 71 | 60.00 | bc,x | 73.33 | abcde,xy | 86.67 | | abc,x |
| NT | NTrNW | 83.33 | ab,x | 87.50 | abcd,x | 91.67 | | abc,x |
| NT | CYS 11 | 44.44 | c,x | 55.55 | de,x | 77.78 | | abc,x |
| NT | CYS 25 | 66.67 | abc,x | 66.67 | abcde,x | 77.78 | | abc,x |
| NT | CYS 55 | 51.85 | bc,x | 59.26 | cde,x | 85.19 | | abc,x |
| NT | CYS 59 | 76.19 | abc,x | 76.19 | abcde,x | 71.43 | | abc,x |
| NT | CYS 71 | 86.67 | ab,x | 93.33 | abc,x | 86.67 | | abc,x |

Each figure is the mean of three replicates. Means followed by the same letter are not significantly different at *P≤0.05* either comparing all treatments in a trial (ab), or within a soil treatment group (xy). Soil treatments are preplant fumigant (PP), after planting or at plant organophosphate (AP), or nontreated (NT). NTrNW is non-transformed Nellie White variety. CYS denotes lines of transformed Nellie White.

## Visual rating of plant growth and health

Plant growth and health was rated visually in all three trials. When rated mid-season this can provide an early indication of how plants will perform at harvest. Poor visual ratings for CYS-11 in both Trial 1 and Trial 2 (which were only significant in the AP soil group treatments) as well as there being very few surviving bulbs at harvest contributed to its not being included in Trial 3.

When rated in July, numerically, plants in Trial 1 were better than NT NTrNW for PP/AP CYS 55, CYS 59 and CYS 71; PP NT4NW, CYS 25, CYS 59, CYS 71; and AP NTrNW and CYS 59. Over all treatments, statistically, there were no differences (*P ≤ 0.05*). Within soil treatment groups, AP CYS 11 and CYS 55 appeared to be less healthy than NTrNW. At harvest, numerically, over all treatments the visual rating of foliage was better than NT NTrNW for PP CYS 25, CYS 55 and CYS 59. There were no significant differences either over all treatments or within soil treatment groups (*P ≤ 0.05)*.

In Trial 2, foliage was visually rated on two occasions, midseason on 3 June 2015 and at harvest. On June 3, numerically, over all treatments visual rating of foliage was better than NT NTrNW for PP/AP NTrNW, CYS 25, CYS 55, CYS59 and CYS 71; PP NTrNW, CYS 55, CYS 59 and CYS 71; AP NTrNW, CYS 25, CYS 55 and CYS 59; and NT CYS 55. Over all treatments compared to NT NTrNW, at *P ≤ 0.05* the visual rating of foliage was better for PP/AP NTrNW, CYS 25, CYS 55, CYS 59 and CYS 71; PP CYS 55; and AP NTrNW. At *P ≤ 0.05* the rating was worse for AP CYS 11. Within soil treatment groups at *P ≤ 0.05* visual rating of foliage was lower than NTrNW for PP/AP CYS 11 and CYS 25; PP CYS 11; and AP CYS 11, CYS 25, CYS 59 and CYS 71.

In Trial 2 at harvest, numerically, over all treatments the visual rating of foliage was better than NT NTrNW for PP/AP NTrNW, CYS 25, CYS 55, CYS 59 and CYS 71; PP NTrNW, CYS 55, CYS 59 and CYS 71; AP NTrNW and CYS 55; and NT CYS 55. Over all treatments compared to NT NTrNW, at *P ≤ 0.05* the visual rating of foliage was better for all treatments in PP except CYS 11; and was worse for AP CYS 11. Within soil treatment groups at *P ≤ 0.05* the visual rating of foliage was lower than NTrNW for PP/AP CYS 11 and CYS 25; PP CYS 11; and AP CYS 11, CYS 25, CYS 59 and CYS71.

In Trial 3, numerically, over all treatments the visual rating of foliage was less than NT NTrNW for PP CYS 25; AP CYS 25 and CYS 71; and NT CYS 25 and CYS 71. Over all treatments compared to NT NTrNW, at *P ≤ 0.05* the visual rating of foliage was greater for PP/AP NTrNW, CYS 55, CYS 59 and CYS 71; PP NTrNW, CYS 55 and CYS 59; AP NTrNW and CYS 55 and for NT CYS 55. Within soil treatment groups at *P ≤ 0.05* the visual rating of foliage was lower than NTrNW for PP/AP CYS 25; PP CYS 25 and CYS 71; and AP CYS 25, CYS 59 and CYS 71.

| Visual ratings of plant growth and health. | | | | | | | | | | | |
| --- | --- | --- | --- | --- | --- | --- | --- | --- | --- | --- | --- |
|  |  | Trial 1 | | | | Trial 2 | | | | Trial 3 | |
| Soil Treatment | Lily | July | | Harvest | | June | | Harvest | | Harvest | |
| PP/AP | NTrNW | 2.67 | abcdefg,xy | 7.67 | a,x | 7.33 | a,x | 8.17 | a,w | 7.83 | ab,xy |
| PP/AP | CYS 11 | 0.89 | g,y | 4.67 | a,x | 1.83 | fghi,z | 1.50 | fgh,y | not tested | |
| PP/AP | CYS 25 | 2.56 | abcdefg,xy | 8.00 | a,x | 4.67 | bc,y | 4.83 | b,x | 4.33 | efghi,z |
| PP/AP | CYS 55 | 3.67 | abc,x | 4.67 | a,x | 6.83 | a,x | 7.00 | a,w | 8.17 | ab,x |
| PP/AP | CYS 59 | 3.56 | abcd,x | 6.33 | a,x | 6.67 | a,xy | 6.83 | a,wx | 7.50 | abc,xy |
| PP/AP | CYS 71 | 3.44 | abcde,x | 4.67 | a,x | 5.83 | ab,xy | 6.83 | a,wx | 6.00 | bcdef,yz |
| PP | NTrNW | 3.56 | abcd,x | 7.00 | a,x | 3.83 | cde,xy | 4.17 | bc,w | 6.00 | bcdef,x |
| PP | CYS 11 | 2.67 | abcdefg,x | 5.33 | a,x | 1.17 | hi,z | 1.50 | fgh,x | not tested | |
| PP | CYS 25 | 4.11 | ab,x | 8.33 | a,x | 1.83 | fghi,yz | 2.17 | efgh,wx | 2.00 | k,yz |
| PP | CYS 55 | 2.89 | abcdef,x | 9.00 | a,x | 4.83 | bc,x | 4.17 | bc,w | 6.17 | bcdef,x |
| PP | CYS 59 | 4.44 | a,x | 7.33 | a,x | 3.50 | cdef,xy | 4.00 | bcd,w | 5.50 | defg,x |
| PP | CYS 71 | 3.22 | abcdef,x | 4.67 | a,x | 2.67 | efgh,xyz | 4.17 | bc,w | 2.67 | ijk,yz |
| AP | NTrNW | 3.22 | abcdef,x | 7.33 | a,x | 4.50 | bcd,x | 3.17 | bcdef,w | 6.50 | abcd,x |
| AP | CYS 11 | 1.33 | fg,z | 4.33 | a,x | 0.58 | i,z | 0.50 | h,z | not tested | |
| AP | CYS 25 | 2.44 | bcdefg,xyz | 7.33 | a,x | 2.83 | defgh,y | 2.17 | efgh,xy | 4.67 | defgh,y |
| AP | CYS 55 | 1.56 | efg,yz | 5.00 | a,x | 3.17 | cdefg,xy | 3.00 | cdefg,wx | 6.33 | abcd,x |
| AP | CYS 59 | 3.00 | abcdef,xy | 7.67 | a,x | 2.17 | efghi,y | 2.00 | efgh,y | 4.17 | fghij,y |
| AP | CYS 71 | 1.89 | cdefg,xyz | 4.67 | a,x | 1.83 | fghi,yz | 1.67 | fgh,y | 3.33 | hijk,y |
| NT | NTrNW | 2.67 | abcdefg,x | 8.00 | a,x | 2.50 | efgh,xy | 2.83 | cdefg,wx | 3.50 | hijk,xy |
| NT | CYS 11 | 1.78 | cdefg,x | 4.67 | a,x | 1.33 | hi,x | 1.33 | gh,x | not tested | |
| NT | CYS 25 | 2.78 | abcdefg,x | 6.33 | a,x | 2.00 | fghi,xy | 1.83 | efgh,wx | 2.33 | jk,y |
| NT | CYS 55 | 2.00 | cdefg,x | 7.33 | a,x | 3.17 | cdefg,x | 3.50 | bcdef,w | 5.67 | cdefg,x |
| NT | CYS 59 | 1.67 | defg,x | 5.67 | a,x | 2.50 | efgh,xy | 2.33 | defg,wx | 4.00 | ghij,xy |
| NT | CYS 71 | 1.67 | defg,x | 4.00 | a,x | 1.50 | ghi,y | 1.83 | efgh,wx | 2.00 | k,y |

Each figure is the mean of three replicates. Means followed by the same letter are not significantly different at *P≤0.05* either comparing all treatments in a trial (ab), or within a soil treatment group (xy). Soil treatments are preplant fumigant (PP), after planting or at plant organophosphate (AP), or nontreated (NT). NTrNW is non-transformed Nellie White variety. CYS denotes lines of transformed Nellie White. Visual rating was on a scale of 1-10 with ten being the highest rating.

## Weight of stems and stem root visual score at harvest

The weights of stems and stem root scores were highly variable. Largest stem weights were associated with PP/AP soil treatment in Trial 2 and with AP soil treatment in Trial 3.

In Trial 2, numerically, over all treatments stem weight per bulb was greater than NT NTrNW for all PP/AP; all PP except CYS 11; all AP except CYS 11 and CYS 59; and all NT except CYS 25. Over all treatments compared to NT NTrNW, at *P ≤ 0.05* stem weight per bulb was greater for PP/AP NTrNW, CYS 55, CYS 59 and CYS 71. Within soil treatment groups at *P ≤ 0.05* stem weight per bulb was lower than NTrNW for PP/AP CYS 11 and CYS 25; AP CYS 11, CYS 55, CYS 59 and CYS 71.

In Trial 3, numerically, over all treatments, foliage weight was greater than NT NTrNW for PP/AP NTrNW and CYS 55; PP NTrNW and CYS 59; AP NTrNW; and NT CYS 55. Over all treatments compared to NT NTrNW, at *P ≤ 0.05* there was no difference in foliage weight. Within soil treatment groups at *P ≤ 0.05* foliage weight was lower than NTrNW for PP/AP CYS 25; and for AP CYS 25 and CYS 71.

In Trial 2, numerically, over all treatments stem root visual score was greater than NT NTrNW for all PP/AP; all PP; all AP and for NT CYS 55 and CYS 59. Over all treatments compared to NT NTrNW, at *P ≤ 0.05* stem root visual score was greater for all PP/AP except CYS 11 and CYS 25; for PP NTrNW and CYS 55; and for AP NTrNW. Within soil treatment groups at *P ≤ 0.05* stem root visual score was lower than NTrNW for PP/AP CYS 11 and CYS 25; PP CYS 11; and AP all except CYS 25. Within soil treatment groups at *P ≤ 0.05* stem root visual score was better for NT CYS 59.

In Trial 3, numerically, over all treatments stem root visual score was greater than NT NTrNW with the exception of PP/AP CYS 25; PP CYS 25 and CYS 71; AP CYS 55; and NT CYS 25, CYS 59 and CYS 71. Over all treatment groups stem root visual score was only greater for PP NTrNW at *P ≤ 0.05*. Within soil treatment groups PP/AP CYS 25, CYS 59 and CYS 71; and PP CYS 25, CYS 59 and CYS 71 had lower stem root visual scores than NTrNW at *P ≤ 0.05.*

| Weight of stems and stem root visual score at harvest. | | | | | | | | | |
| --- | --- | --- | --- | --- | --- | --- | --- | --- | --- |
|  |  | Stem weight (grams) | | | | Stem root visual score | | | |
| Soil treatment | Lily | Trial 2 | | Trial 3 | | Trial 2 | | Trial 3 | |
| PP/AP | NTrNW | 41.39 | a,x | 212 | abcd,xy | 7.11 | a,x | 9.67 | a,x |
| PP/AP | CYS 11 | 8.50 | defg,z | not tested | | 3.17 | cdef,y | not tested | |
| PP/AP | CYS 25 | 16.02 | cde,yz | 91 | cdef,z | 3.43 | cdef,y | 4.33 | cd,y |
| PP/AP | CYS 55 | 24.68 | bc,xyz | 227 | abcdxy | 5.45 | ab,xy | 7.00 | abc,xy |
| PP/AP | CYS 59 | 32.38 | ab,xy | 121 | bcdef,yz | 5.64 | ab,xy | 6.33 | abc,y |
| PP/AP | CYS 71 | 41.38 | a,x | 166 | abcdef,xyz | 5.36 | ab,xy | 6.00 | bc,y |
| PP | NTrNW | 14.24 | cdef,xy | 212 | abcd,xy | 4.02 | bcd,x | 9.00 | ab,x |
| PP | CYS 11 | 1.67 | fg,y | not tested | | 1.66 | efg,y | not tested | |
| PP | CYS 25 | 9.91 | defg,xy | 76 | def,xy | 2.51 | defg,xy | 3.67 | cd,y |
| PP | CYS 55 | 17.23 | cde,x | 106 | cdef,xy | 4.50 | bc,x | 7.00 | abc,xy |
| PP | CYS 59 | 14.89 | cde,xy | 257 | ab,x | 3.16 | cdef,xy | 5.00 | cd,y |
| PP | CYS 71 | 19.27 | cd,x | 45 | f,y | 3.08 | cdefg,xy | 4.00 | cd,y |
| AP | NTrNW | 14.79 | cde,x | 272 | a,x | 4.02 | bcd,x | 7.00 | abc,x |
| AP | CYS 11 | 1.50 | g,z | not tested | | 1.67 | efg,y | not tested | |
| AP | CYS 25 | 12.07 | defg,xy | 151 | abcdef,y | 2.53 | defg,xy | 5.67 | bc,x |
| AP | CYS 55 | 8.52 | defg,y | 181 | abcdef,xy | 1.71 | efg,y | 3.67 | cd,x |
| AP | CYS 59 | 6.14 | efg,yz | 166 | abcdef,xy | 1.89 | efg,y | 4.67 | cd,x |
| AP | CYS 71 | 7.70 | defg,yz | 106 | cdef,y | 1.96 | efg,y | 5.00 | cd,x |
| NT | NTrNW | 7.37 | defg,xy | 197 | abcdef,x | 1.61 | fg,y | 4.33 | cd,x |
| NT | CYS 11 | 11.00 | defg,xy | not tested | | 1.33 | g,y | not tested | |
| NT | CYS 25 | 5.85 | efg,y | 45 | f,x | 1.33 | g,y | 2.00 | d,x |
| NT | CYS 55 | 10.73 | defg,xy | 212 | abcd,x | 1.76 | efg,y | 4.67 | cd,x |
| NT | CYS 59 | 15.89 | cde,x | 136 | abcdef,x | 3.11 | cdefg,x | 3.67 | cd,x |
| NT | CYS 71 | 7.58 | defg,xy | 60 | ef,x | 1.33 | g,y | 3.67 | cd,x |

Each figure is the mean of three replicates. Means followed by the same letter are not significantly different at *P≤0.05* either comparing all treatments in a trial (ab), or within a soil treatment group (xy). Soil treatments are preplant fumigant (PP), after planting or at plant organophosphate (AP), or nontreated (NT). NTrNW is non-transformed Nellie White variety. CYS denotes lines of transformed Nellie White. Visual rating was on a scale of 1-10 with ten being the highest rating.

## Number and weight of bulblets

The number and size of bulblets that develop on stems belowground is important because they are often utilized in subsequent plantings. Trial 1 conducted May-September 2014 was planted directly from the tissue cultured plantlets received from USDA. This was too short a period of time to produce bulblets, so this data is not available in the table. Planlets not used for Trial 1 were raised in the greenhouse until used for Trial 2. The plants in Trial 2 were in the field for a full growing season, developed bulbs at the base of the plant that were used for Trial 3, and produced bulblets on the underground stems. Both number and weight of bulblets generally increased from Trial 2 to Trial 3 correlated with the greater size of bulbs. The largest number and weight of bulblets in Trial 2 were associated with the PP/AP soil treatment.

In Trial 2, numerically, over all treatments number of bulblets that developed on a plant was greater than NT NTrNW for all PP/AP; all PP; AP all except CYS 11 and CYS 59 ;and all NT except CYS 71. Over all treatments compared to NT NTrNW, at *P ≤ 0.05* the number of bulblets was greater for PP/AP NTrNW, CYS 55, CYS 59 and CYS 71; and for NT CYS 11 and CYS 59. Within soil treatment groups at *P ≤ 0.05* the number of bulblets was greater for PP CYS 55 and CYS 59; and NT CYS 59.

In Trial 3, numerically, over all treatments the number of bulblets was greater than NT NTrNW for PP CYS 55, AP CYS 55 and CYS 59; and NT CYS 55. Over all treatments compared to NT NTrNW, at *P ≤ 0.05,* PP CYS 71 had fewer bulblets. There were no significant differences within soil treatment groups.

In Trial 2, numerically, over all treatments the weight of bulblets per plant was greater than NT NTrNW for all PP/AP; all PP all except CYS 11; all AP except CYS 11 and CYS 59; and all NT except CYS 71. Over all treatments compared to NT NTrNW, at *P ≤ 0.05* weight of bulblets per plant was greater for PP/AP NTrNW, CYS 55, CYS 59 and CYS 71; and PP CYS 55. Within soil treatment groups at *P ≤ 0.05* weight of bulblets per plant was greater than NTrNW for PP CYS 55 and lower for NT CYS 59.

In Trial 3, numerically, over all treatments the weight of bulblets was greater than NT NTrNW for AP NW, CYS 55 and CYS 59; and NT CYS 55. Over all treatments compared to NT NTrNW, at *P ≤ 0.05* weight of bulblets was less for PP/AP CYS 25 and CYS 59; PP CYS 25 and CYS 71; and AP CYS 25. Within soil treatment groups at *P ≤ 0.05* weight of bulblets was greater than NTrNW for PP/AP CYS 55.

| Number and weight of bulblets per plant. | | | | | | | | | |
| --- | --- | --- | --- | --- | --- | --- | --- | --- | --- |
|  |  | Number of bulblets | | | | Weight of bulblets (grams) | | | |
| Soil Treat-ment | Lily | Trial 2 | | Trial 3 | | Trial 2 | | Trial 3 | |
| PP/AP | NTrNW | 3.26 | abc,x | 4.80 | abcd,x | 10.38 | b,xy | 23.20 | def,y |
| PP/AP | CYS 11 | 2.83 | abcde,x | not tested | | 4.01 | cdefg,y | not tested | |
| PP/AP | CYS 25 | 2.49 | abcde,x | 4.13 | abcd,x | 6.11 | bcdef,y | 16.00 | f,y |
| PP/AP | CYS 55 | 3.32 | ab,x | 6.00 | abc,x | 9.78 | bc,xy | 36.27 | bcde,x |
| PP/AP | CYS 59 | 4.13 | a,x | 4.00 | bcd,x | 17.17 | a,x | 16.80 | f,y |
| PP/AP | CYS 71 | 4.16 | a,x | 4.60 | abcd,x | 18.80 | a,x | 22.93 | def,y |
| PP | NTrNW | 1.20 | efg,y | 5.00 | abcd,x | 3.17 | defg,yz | 20.67 | ef,x |
| PP | CYS 11 | 1.33 | defg,y | not tested | | 1.06 | efg,z | not tested | |
| PP | CYS 25 | 1.93 | bcdefg,xy | 3.47 | cd,x | 4.00 | cdefg,xyz | 15.07 | f,x |
| PP | CYS 55 | 2.73 | abcde,x | 5.87 | abc,x | 7.92 | bcd,x | 24.00 | def,x |
| PP | CYS 59 | 2.67 | abcde,x | 5.13 | abcd,x | 6.72 | bcde,xy | 28.13 | cdef,x |
| PP | CYS 71 | 1.82 | bcdefg,xy | 2.47 | d,x | 5.02 | bcdefg,xyz | 13.47 | f,x |
| AP | NTrNW | 1.97 | bcdefg,x | 5.27 | abc,xy | 5.42 | bcdefg,x | 39.87 | bcde,xyz |
| AP | CYS 11 | 0.50 | fg,x | not tested | | 0.42 | fg,x | not tested | |
| AP | CYS 25 | 1.60 | bcdefg,x | 3.67 | cd,y | 2.80 | defg,x | 17.60 | f,z |
| AP | CYS 55 | 1.50 | cdefg,x | 6.80 | a,x | 2.90 | defg,x | 44.27 | abc,xy |
| AP | CYS 59 | 0.57 | fg,x | 6.60 | ab,x | 1.35 | efg,x | 60.80 | abc,x |
| AP | CYS 71 | 1.53 | bcdefg,x | 3.93 | bcd,y | 4.76 | bcdefg,x | 23.60 | def,yz |
| NT | NTrNW | 1.17 | efg,yz | 6.00 | abc,xy | 2.06 | efg,xy | 37.73 | bcde,xy |
| NT | CYS 11 | 3.00 | abcd,xy | not tested | | 2.83 | defg,xy | not tested | |
| NT | CYS 25 | 2.12 | bcdef,xyz | 4.07 | abcd,xy | 3.45 | defg,xyz | 20.53 | ef,y |
| NT | CYS 55 | 1.58 | bcdefg,xyz | 6.67 | ab,x | 5.13 | bcdefg,xy | 47.33 | abc,x |
| NT | CYS 59 | 3.28 | abc,x | 4.93 | abcd,xy | 6.78 | bcde,x | 31.60 | bcdef,xy |
| NT | CYS 71 | 0.25 | g,z | 3.33 | cd,x | 0.00 | g,z | 24.13 | def,xy |

Each figure is the mean of three replicates. Means followed by the same letter are not significantly different at *P≤0.05* either comparing all treatments in a trial (ab), or within a soil treatment group (xy). Soil treatments are preplant fumigant (PP), after planting or at plant organophosphate (AP), or nontreated (NT). NTrNW is non-transformed Nellie White variety. CYS denotes lines of transformed Nellie White.

**
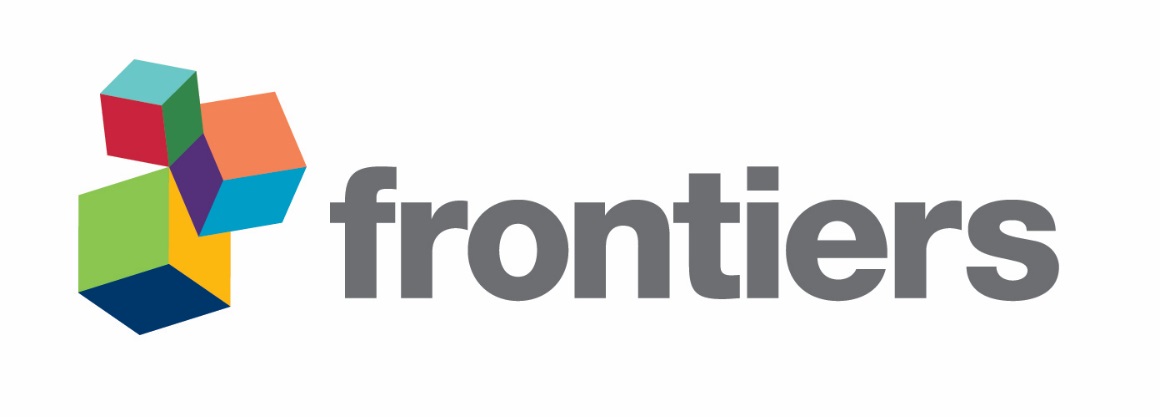
**
